# Supplementary material for: Kozak Similarity Score Algorithm Identifies Alternative Translation Initiation Codons Implicated in Cancers
Source: Int J Mol Sci. 2022 Sep 12;23(18):10564. doi: 10.3390/ijms231810564 (PMC9506484; doi:10.3390/ijms231810564)
Supplement: Supplementary file 1 [file ijms-23-10564-s001.zip › Supplemental File S1-Predicted Upstream TICs Identified in Cancer Genes.pdf]

Supplemental File 1. Predicted Upstream TICs Identified in Cancer Genes.

Below are the 5'UTRs of mRNAs from genes overexpressed in cancer. Codons colored in red have KSSs ≥ 0.8. Upstream ATGs that may be used for translation are bolded. Potential alternative TISs are followed by their KSS score. NCBI Nucleotide accession numbers for each sequence are in parentheses.

EPHA4 (NM_001304536.2)

ACGCGTGCTCATCTTGTGTAAAAGTAAAAGTTGCTCATGTCATTAGCTCGGCTCTGAGTTTGCATGAGA
sAACCAGCGCAGCTCTCGAGCCACGGGAGAAAACACGATCTGCTGCGGAGCCCAGTGACCTGAAACT
GTAGCAGTGACTCGAGACCTGTCCTCTCCTCGACGTCGCCCTATAACCCTCCCGCTTTATTTAGGATCC
GCTTTTCCGAGAGCAGCCACTCAGGCAACCCCCGGAAGAAGCGGCAGGAGCAGCGTTG(0.82)GCACC
GGCGAACC

YY1 (NM_003403.5)

CTCCCTCTGCCTTCCTTCCCCACGGCCGGCCGCCTCCTCGCCCGCCCGCCCGCAGCCGAGGAGCCGAG
GCCGCCGCGGCCGTG(0.91)GCGGCGGAGCCCTCAGCC

ERBB2 (NM_004448.4)

CCCCTCCATTGGGACCGGAGAAACCAGG(0.87)GGAGCCCCCGGGCAGCCGCGCGCCCCTTCCCACG
GGGCCCTTTACTGCGCCGCGCGCCCGGCCCCCACCCCTCGCAGCACCCCGCGCCCCGCGCCCTCCCA
GCCGGGTCCAGCCGGAGCC**ATG(0.86)**GGGCCGGAGCCGCAGTGAGCACC

FOX2 (NM_001031695.4)

CTTCAGCCATCTGCCTGAGATATGGGAGGGAGCTGGAGAAAGAAG(0.81)GAGGGGGAGAGACTGCC
AGAGAGGAAGAGAAAAGAAAGGAAGAAACATTAGAAAGAAAAAGGAAGGAAAACGGTATAAAGAG
AGATCAATTACCCACCCTTAAATAGCTAGATTGGGGGGGGAGGGGGGTGGAAAAGAAAGCTGTGGA
GGTGTGCCCCAGCACG(0.88)GCTGCTTTGAAAGGTTTATCATCTATCCGTTTGGTTT

KIT (OM304357.1)

TCTGGGGGCTCGGCTTTGCCGCGCTCGCTGCACTTGGGCGAGAGCTGGAACGTG(0.82)GACCAGAGC
TCGGATCCCATCGCAGCTACCGCG

AXL (NM_021913.5)

GAGAAGGCGGCTGCTGGGCAGAGCCGGTGGCAAGGGCCTCCCCTGCCGCTGTGCCAGGCAGGCAGT
GCCAAATCCGGGGAGCCTG(0.87)GAGCTGGGGGGAGGGCCGGGGACAGCCCGGCCCTGCCCCCTCC
CCCGCTGGGAGCCCAACAACTTCTGAGGAAAGTTTGGCACCC

MYC (NM_002467.6)

AACTCGCTGTAGTAATTCCAGCGAGAGGCAGAGGGAGCGAGCGGGCGGCCGGCTAGGGTG(0.80)GA
AGAGCCGGGCGAGCAGAGCTGCGCTGCGGGCGTCCTGGGAAGGGAGATCCGGAGCGAATAGGGGG
CTTCGCCTCTGGCCCAGCCCTCCCGCTGATCCCCCAGCCAGCGGTCCGCAACCCTTGCCGCATCCACG
AAACTTTGCCCATAGCAGCGGGCGGGCACTTTGCACTGGAACTTACAACACCCGAGCAAG(0.84)GAC
GCGACTCTCCCGACGCGGGGAGGCTATTCTGCCCATTTGGGGACACTTCCCCGCCGCTGCCAGGACCC
GCTTCTCTGAAAGGCTCTCCTTGCAGCTGCTTAGACG

MET (NM_001127500.3)

AGACACGTGCTGGGGCGGGCAGGCGAGCGCCTCAGTCTGGTCGCCTGGCGGTGCCTCCGGCCCCAA
CGCGCCCGGGCCGCCGCGGGCCGCGCGCGCCGATGCCCGGCTGAGTCACTGGCAGG(0.82)GCAGCG
CGCGTGTGGGAAGGGGCGGAGGGAGTGCGGCCGGCGGGCGGGCGGGGCGCTGGGCTCAGCCCGGC
CGCAGGTGACCCGGAGGCCCTCGCCGCCCGCGGCGCCCCGAGCGCTTTGTGAGCAGATGCGGAGCC
GAGTGGAGGGCGCGAGCCAGATGCGGGGCGACAGCTGACTTGCTGAGAGGAGGCGGGGAGGCGCG
GAGCGCGCGTGTGGTCCTTGCGCCGCTGACTTCTCCACTGGTTCCTGGGCACCGAAAGATAAACCTCT
CATA

HP (AF026219.1)

CCCAGCCAGGAC**ATG**(0.81)GCCGCACCTCTCCTCATCAGGAGCGCCGGCTCACGGACTTCTCGCCCA
ACTCCCTGAGCGCTCCCTCGTTTCGATCTTTAGAAAACCCTGCTTTCTTTCTGGGGCCGTGACGAGG(0.8
2)GGCAGGGAGCGGCGAGCAAG(0.90)GATGCGTTGAGGACCGCGAGGGCGCGCGTCTCGGGTGCCG
CCGTG(0.86)GGTCCCGACGCGGAAGCCGAGCCGCCTCCGCCTGCCTCGACTTCCCCACAGCGCTTCC
GCCGCCGCCTGCCGTGCTTGATGTGCAGAAAGAAGCCGGACACC

EPHA1 (NM_005232.5)

GCAACCTG(0.80)GCGCTGCCATCCGGGCCACTGTCCCAGGTCCCGGCCCGGAGCT

MYCN (NM_001293228.2)

AGGCTGTGACAGTCATCTGTCTGGACGCGCTGGGTGGATGCGGGGGGCTCCTGGGAACTGTGTTGGA
GCCGAGCAAGCGCTAGCCAGGCGCAAGCGCGCACAGACTGTAGCCATCCGAGGACACCCCCGCCCC
CCCGGCCCACCCGGAGACACCCGCGCAGAATCGCCTCCGGATCCCCTGCAGTCGGCGGGAGGTAAG
GAGCAGG(0.83)GCTTGCAAACCGCCCGGCGCCCAGGGAAGCGACGAGCGCCGGGGCAAGGCAAGC
CCTG(0.80)GACGGGATT(0.82)GCGACGTGCGCACCGGGCGCCCTAATATGCCCGGGGGACTGTTTCT
GCTTCCGAAACAAAACCATCTCTGGGTTTTCCCAGAAAAGCCAGTTCCAGCCCCGAAGGCATCCTG(0.
87)GCTAGAGGAGACCCGCCCTAATCCTTTTGCAGCCCTTACCGGGGGGAGTAATGGCTTCTGCGAAA
AGAAATTCCCTCGGCTCTAGAAGATCTGTCTGTGTTTGAGCTGTCGGAGAGCCGTGTTGGAGGTCGGC
GCCGGCCCCCGCCTTCCGCGCCCCCCACGGGAAGGAAGCACCCCCGGTATTAAAACGAACGGGGCG
GAAAGAAGCCCTCAGTCGCCGGCCGGGAGGCGAGCCG

FOXM1 (NM_202002.3)

AACGCTCCGCCGGCGCCAATTTCAAACAGCGGAACAAACTGAAAGCTCCGGTGCCAGACCCCACCCC
CGGCCCCGGCCCGGGACCCCCTCCCCTCCCGGGATCCCCCGGGGTTCCCACCCCGCCCGCACCGCCG
GGGACCCGGCCGGTCCGGCGCGAGCCCCCGTCCGGGGCCCTG(0.84)GCTCGGCCCCCAGGTTG(0.85)
GAGGAGCCCGGAGCCCGCCTTCGGAGCTACGGCCTAACGGCGGCGGCGACTGCAGTCTGGAGGGTC
CACACTTGTGATTCTCAATGGAGAGTGAAAACGCAGATTCATA

KPNA2 (NM_001320611.2)

GTTGACTAGGCCTCGGGGGCGACGTTCATTGACCAGGCGGCTGAGTTTCGCGGGGTCTGCGGGTTTA
GGGCGCCGACGCTCTGCAAACGGCAGCGGAGGCCTTAACGCGTCGCGGCCGGGAGAATCGGAGCGA
GCCAGGCTTGAGGGCGAATGTCCCGGGAGGACTTGTGGCGGCCCTG(0.81)GTTTCGCCTCCTCCTTTC
TCGTTGCATCTTCCCCGGCGTG(0.83)GGGGCAGGGGCTGGGGGTGGCAGTTGGGAGCACCGTCTCTG
ACACCTAGGCCCCGGGGGTGGCTCTGCCACCAAG(0.84)TCGTAGGCGAGCGTAATGAAAGCAAAGAT
AGCAAATTGTAAGGAGGGCTTTGACTTTTGTCATCTTCTGAATGACGCACAGCTGATGTCATCCTAATT
AAGCGCATTCACTCCTAGTCTCTGGAAATGCAAATCAAATATGCGAGATGCGGCACTGCATTTTTAATG
TTGACCATAATGTAATGATGAAGCAAATTTTTATTTAATTTTCGAGTCTGCACCTATTTTGGCCAAGTAA
AGACAGTAAAATAGGGAGAGCCTCCTGAGGATCTGAATTTATTGCCCTGGCACATAATTTTTAGCTCTT
TCTCCCTTTGTCTCATAACC

STAMBP (NM_006463.6)

GTTTCCGGAACCTCCGGGTGTCATCCGCGGGGAAAGGTGGGGAAGGGTCCCGGGAACGTG(0.88)GTG
GGGCAGG(0.80)GCCTCCGAGCGTGGTTGGACTTTGAAGGGGATCGGCCGCCATGCTGCATCCCCTTTT

TGGAATTGCTCAACCAGGTGGTAACCGGCGCCGCTTCCTGGCCTTGGGAGGTGGTTCCTTTCTTAACCC
ACAAGAACCTCTCCCAAGAGAACTTGGTCCTG

MAP3K8 (NM_005204.4)

ACTCGTCCGCTCCGCTCTGGACTGCGCGCCACGCTCTGGGGTCCGGCGCCCTG(0.82)GTTCCTGCTTCT
GCCGCTGCCGCCGCCGGATCCCAGTGGCCCGGCGTGCTCGGCTCCCACAGGCCTGCAGCCAGCATC(9
0)GCACCGAACCTTCGGGGGGCCGCGGCTGGAGCGCTCGGCCGGCGTG(0.83)GGAGCGCCAAG(0.85)
GCCGCAGATGCAATCTTCTTACCGCGAAGAAGCCAGGGGAATAGGTAGCCACATCTTGTTTGCAGATA
AGAAAGGAAGCTAACGCAGTATCTGCAAAGCCAGGAGTCTGACTCAGTACTTTTCTCACTCATGCATA
CAAAGCAGCTAAAAATGACACAGCTTATTTACCATGCCCCTGACACTGCACTGAGCACTTTATGAGCTT
GAACTCTGTTAATCCTCACGACCACCTCATGAGACTCTCCAGAAAGAGCAACAGTA

CDKN3 (NM_005192.4)

ACCGGTGAGTCGCCGGCGCTGCAGAGGGAGGCGGCACTGGTCTCGACGTG(0.80)GGGCGGCCAGCG

ADAR (NM_001111.5)

GAACCGGAGCCATCTTG(0.88)GGCCCGGCGCGCAGACCCGCGGAGTTTCCCGTGCCGACGCCCCGGG
GCCACTTCCAGTGCGGAGTAGCGGAGGCGTG(0.81)GGGGCCTCGAGGGGCTGGCGCGGCCCAGCGG
TCGGGCCAGG(0.80)GTCGTGCCGCCGGCGGGTCGGGCCGGGCAATGCCTCGCGGGCGCA

RCC1 (NM_001048194.4)

AGAGGCCTGCAGAGCGCATGCTCTGGGGCAGTTCGCGGCCCGGCGGGGAGCGCCGGAGTTCCTTGT
GGCCGACGTGCACCAAG(0.85)GACAGGAAG

COL11A1 (NM_001854.4)

ACTGACGGCATGAAGCCTTTAGGGGCACACAGTACTCTCAGCTTGTTGGTGGAAGCCCCTCATCTGCC
TTCATTCTGAAGGCAGG(0.81)GCCCGGCAGAGGAAGGATCAGAGGGTCGCGGCCGGAGGGTCCCGG
CCGGTGGGGCCAACTCAGAGGGAGAGGAAAGGGCTAGAGACACGAAGAACGCAAACCATCAAATTT
AGAAGAAAAAGCCCTTTGACTTTTTCCCCCTCTCCCTCCCCAATGGCTGTGTAGCAAACATCCCTGGCG
ATACCTTG(0.85)GAAAGGACGAAGTTGGTCTGCAGTCGCAATTTCGTGGGTTGAGTTCACAGTTGTGA
GTGCGGGGCTCGGAG

BUD31 (NM_003910.4)

GTCGAGAAGCAGCTACCCAAGCTCCAGGAGCTTCCGGTATGTGTTTTCCCTCTGTTCTCGATTACCTTG(
0.86)GCAACGGCTGAGGCGGGAGACCGGTGGTCTGCACCGTCCTG(0.80)GAGGGAGATATGAGTGGC
TGGACTCTCAGCCAGCCACTGGGATGTGTTCGGGCTTTGGACCTTGAGGCCGGAGAGAGCTCCCGAG
AGGAGGCGGCGCCACGTTCGTTCTTCTGAGGGGACGGTAGATTTGGGGGTTTTCCTCTAGGATTCTCG
CGCCGTTTCCTCTGAAGAAACAGGACCAGAGAGGGAAGGTGACCTGAAAGTCACAGAATAATTTTTTA
GAGCTGAACAAGAATCCAAGCCTGCAACTGCAGAGACGAGAGATCTTTCTGCTGTCTATACTCTTGGA
AAGCACATCCTAAGATCTTTGCAGATTATCCTGTGGAAGGAAA

KCNH2 (NM_000238.4)

AACCCTTCCGCGGCCGGGCCGAGCCGCAGGCGCTGCCCGAGCCGCGGGCGCTGGAGCGGCTGTCGG
CGCGGTG(0.82)GCAGGCGGGAGCCAGGAGTCCGGGGCTCGGGCGGGCGGAGCGCAGCGCGGGGAC
GCGGCGGAGCGGGCCCCGCGGCTCGGCGGGGGCCGGCAGACAGGTGTGCCGGCGGCGGGCGGCTC
GCGCTCAGGCGGCTCCAGGCCGGGCCCGGGGTCCGGAGCGGGGAGCCGGGAGCCGAGCGAGGACC
CGGCGCCCGCAGTCCAGTCTTG(0.82)GCCGCGCCCGTGCTCGGCTTG(0.86)GCCGCGGGGTGCGGGG
ACCACG(0.91)GCCCGGCCGGGCCACCCGAAGCCTAGTGCTGGGCCGGGCCGGGCCGGGGTGGGTGG
GGGCCCGCCCGGCCCGCCCATGGGCTCAGG

BCAP31 (NM_001139457.2)

GATGGGCCTCCGGGACGGTGTGCCAGGCCGGGGCCAAGTCGGAGGCCCCTCGCTCTGGGTGGGCGC
TGGGGCCCGCGAGG(0.80)GCTACTGTAAGGACCCCTGGCTTCTGAGGATACTGCGTCTAGAACTTTCT
CCGTATGGGGCCTTGAGGTGCTTGGTCGAGACCTGCCTTTGCGCTTGGTCCCGAATCCTGCCCTCTAG
GAGTCGCTCTTGCGGGCCTCCAGCCCACCGGAGGCGAAGCGGCCCCGGGCGGAAGGCCGCTGGATC
CTCGAGGGAGGTGCCGGTTTCTCTCCGCGGGCGCCGTG(0.89)GGGACGGTG(0.82)GGAGGCGGGGG
CGTCGGCAGCGCTTGGACTAGGTGCGGCCTTG(0.82)GGCCTGCCTGGTAGCGGGGATTTGGGCCCGC
AGAGCGCCCGCCTCTGCGGCTGAGTTCTGCCTGGCGGGGAAGGGAGCGCCCG

NME1 (NM_198175.1)

GCAGAAGCGTTCCGTGCGTGCAAGTGCTGCGAACCACGTGGGTCCCGGGCGCGTTTCGGGTGCTGGC
GGCTGCAGCCGGAGTTCAAACCTAAGCAGCTGGAAGGGCCCTGTGGCTAGGTACCATA(0.84)GAGTC
TCTACACAGGACTAAGTCAGCCTG(0.86)GTGTGCAGGGGAGGCAGACACACAAACAGAAAATTGGAC
TACAGTGCTAAGATGCTGTAAGAAGAGGTTAACTAAAGGACAGGAAGATGGGGCCAAGAG

RPA3 (NM_002947.5)

AAGCTACTCAGATAAGAGGCTCCAAGAGGACATTTTTGGATGTGAAAAACAATGAGAAGGAGGACAA
CACACATTTACAATCGTCTTAATTTTGTACTCAGAAAAAGGATGTGAAGACAATGCACAGGGAATACA

ATAGTTTCAGATCTGTGTACAGTTTCCTTTTGCTTCATCTCCTGCAACAATGTAATGAAGACACCATGAT
ATCATTAACATTTCACACAAAAGGAAAATGAGGCTGAAATGGTGTGGGCAAG(0.80)GCCCAGGAATCT
GGAGCATCCCTAACCAAGCAGGAGAGCACCTGGGATAGAGAAAGTGCTCAAGAATGTTCACTTACTG
ATTACTACAATCAAAAAAAGATACGACACTAATTTACCACATTCTTCTTACTTATTTTATGAGATACTATT
CTTCCAAGGTG(0.81)GAGAAAGTGGAGAAAGTAGAGTGACGCAGCTAAGGGAGTAAATCGACCCTCA
GCCAACAAGTGGCAAAAGCCTGAAGAAAGTGATCAAGATCACTGATGACCCCGCTGCCCATCTCCAA
GGGGGCGGGTATCACAACCCCGACGCCACACCACGTATCATTCCGCAAAACTCCCGCGCCTCCCACG
CAGAACTGGCAAGAGG(0.82)GAAGGCGAGACAGCAGTGAACAGCTGGTACGCAGCACCCACAGCAC
CGCGGCAGCAGCTAGTGCCGACTCCCGCCTAGCTCTTTTGACTCTGTTCGCGGGAAGAATGGGGAAAC
AGTAAGGTTGCGGCGCCTCCCGCGAGACGAGGTACCTGAGGCTG(0.80)GCCCCGCAGTCCCCCGCCG
CACCAGCACCGGAGCTTCACACCCCACTTCCGGGGTCAAGTCACCGCCGGGAATCCTGTGATCGCAG
AAAGGTAGTCTCAGGTTCCGCCCCTATCCAAGTCCCGCCTCCACTGCCTCTCGCCCTGTATCTGTCAAC
TTCCGGGACGCCGCGCGTCACTAAGCAGCCAATCTCCACTTCCGGACTCATCCAGCCCCTTCTCCACC
CCTTTCAGAGACAGCGCGATT(0.81)GCGATTTAGGTTTCCGCGCATTTAATTGGCGAAGCTG(0.81)GA
GCGCTAGTCTTCGCTGATTGGTGCCGAGAAATCTGCCCCATAGACACCCGCGGGGCGCACAGTTTCAG
TCGTCCGTGGGTTTCCCGCCAGCCGCAGTCTTGGACCATAATC

ELOC (NM_005648.4)

CTTTCGACTGCCCCGGAACCCACCGGAGCAGGCAGCTGGGGGTGGGGGGGCGGCCCTG(0.81)GGAT
AGGGGCTGTGGCAGTACGCGGGGACCCGGCTGCGGTG(0.81)GCTGCGGGACTGACGAGAAACTACT
AAAGTTCCTGGGGAAGCAAAGTAGAATTTCATAAGAACAAA

DRAP1 (NM_006442.4)

GGGCGGCGAGCAGGCCCGGGAGCCGGGAGGCTGCGGGCGGCGGCGCTG(0.81)GACCCGACGCGGC
GAGAGAGGCCCCGAG

UBE2S (NM_014501.3)

GGCTCAGTGCTGCCGGGCACCGGGGCGGCGGGTTGGTCTACGCTGTGCGCGGCGGACGTCGGAGGC
AGCGGGGAGCGGAGCGGGGCCGCCGGGGCCTCTCCAGGGCCGCAGCGGCAGCAGTTGGGCCCCCC
GCCCCGGCCGGCGGACCGAAGAACGCAGGAAG(0.83)GGGGCCGGGGGGACCCGCCCCCGGCCGGC
CGCAGCC

PSMB8 (NM_004159.5)

AAACTCCGCAGTGCTCAGCCAAGCAGG(0.81)GAGCAACGCTAGGAAGGGCGGGCAGAAAG(0.80)GG
CACGCTCTTGTGGGTGACTACAGGTTAGGAGACCGTTGAACCTG(0.85)GAGGGGCCCTAGG**ATG(0.8**

0)GACCCCGTGGAAAGATTCAGAGACTGCGCCCTCTCCCTGGCGCCGCCTTCCCCTACACGCGGCGGG
TATATTCTGTTGCAGTTGGCCCAGGACCTGTTTCCAAGACTCTGCCCCCTCGCACTTCCGTCCCTCCTGG
TTTTGTAAAGTG

RNPS1 (NM_006711.5)

GGAGGTGTCGAGGCCGTG(0.84)GCCCGGAAGTGGTCGGGGCCGCTGCGGACGGAAGGGCGCCTGTG
CTTCGTCCGCTCGGCGGTGGCCCAGCCAGGCCCGCGGGACTCAGACCAGCGGGGAGCGCGGCCTCC
GCCCTTGGGGCCCTCCCGCCGGGCCGGAGACCCAAGCCCCCAACGCCAGGCCCTGCCCTG(0.83)GAA
GCGCTCGCGGCCCGGCGCCTGGACGGGGGAGTTGCTGCTCTTTGGCGTAAATTGCAATCGATTAGGG
ATCGTTTCTCAGAATCAAGTTAGAAGTGAGAGTTCAGATAAGTGAGGCCGCCATT(0.88)GCTGCTTTGA
ACACCTCAGAAGGGGAGA

DLGAP5 (NM_014750.5)

AAGTTTGAATTTCGTGGAGGCTCGGGTTGTGAGGGTTCCTGCTTCGGAGTCGGCGGTGGTCGTCCAGA
CCGAGTGTTCTTTACTTTTTGTTTGGTTGAGGTTTCACGCTAGAAGGTG(0.80)GCTCAGG

MFAP2 (NM_017459.3)

GCAACTTGGTCTCACAGTG(0.80)GCTTAGGCCAGGGTGGGAGCAGTGAACGGAGTCACAAAAGAAAT
TTTTCAGCTGTCCTCTCTGACACCACCCCGGCCTGCCTCTTTGTTGCC

USP7 (NM_003470.3)

GACATTTCACGCCGCCGCCATTTTGAGAGCGAGCCGAGCCGAGCTGCCGGGCGCCGCGTCCGCTGCC
GGAGCCCCGACGACG(0.80)ACGCCGAGGAGGCGGAGGCCGCGGCTCTCGGAACGCGGCCGGCCCG
TCGCCCGCCCGCTCCGCCGCTCCCGCCGGCCCCAGGGCCCGCAGGCCCGCCGCGGCCGGCCAGGCC
TCCCGTCCGCCGCGCCCGGCCCGAGGCGGGGCTGACTCCGCGGCCCCCGACCGGCCGCCCTCCGCC
CCCGGCCGGCCCGCGGCCCCGCAGCCCCGGCCCCGGCCCCGGCGGCGGGAGGCGGGCCCCGCGGC
GGCGGCGGCGGCGGCCGCAGCGAGCGACGAGGCCGCCCGGCCCGGCCCGCCGGCCGCCCGCCCGC
CTCGGCGCCGAGATT(0.80)GGGCGAATCGCGAGCAAGTACGTGCGCGTCTCCCTGCCGCCGCCGCCG
CCCGCCGCGGGCCGCCCCGGGGCCGCCGTCGCCGACGACGCGCGGGAGGAGGAGGAGGAGGCCGC
CCCGCCGCCGCCGCCGCCGCCGCCGCCCCGGCTCGCCGCCGCCCGCCCGCCGGGCTCGCAGCCCCG
GCCCCCGGCCGCAGGCGAGGCCCAGGCCGCGGCCGAC

LAGE3 (NM_006014.5)

CTCGCGGGGTGCGCCTCTGGGATAGGCGACCACG(0.85)GTGTCTTCAAAAGCCCCGTCAGGGTTGGC
TTCCTGGGGCCGGACCGACTGTGGGTCAGTTTGCACCAGCGCTCTGGAATCGAGTTACGCGCGAAAG

GGCAGAGTTTCTGGAGGAAACCGCAGCCTCTCAACCGCTGACCGGGTCTCAGAAGGCCCCGGCAGG(0.86)GCCGCTTGGCGGGAACTGACCACGCGCCAGTCAGGCTCTCCAGGGACCTGCGCAGGCGCGTGTGGGCGGAGTCGTGCGCAGGGGGCGGGGCTTCGGGAAGGAGCCACAGAGAGG(0.80)GCGGGGCGTAGGACCTGCGCTTCGGGGGTGGAGTCGGAGCGGCGCGGCGGCGGTC

NUTF2 (NM_005796.3)

GCAGACCGCGCTGGGTTGCCGCTGCCGCTGCCGCCATC(0.86)GTGCCAGCCCCTCGGGTCTCCGTGAGGCCGGGTGACGCTCCAGA

TRIM28 (NM_005762.3)

AGTGACGCAGAGGCTGGAGACGACTCTACGGCGGCGAAGAGACGCGGGTTGAGGAAGAGGGACGGATTGCCCATGCGCTTGGGCGCACAGCGGCCCGCTTCTGTGTGGTCTGGAGGTGGAGCTGAGAGGGGAATCACACTCTATAAAGGTTCGCATACCCCACTGGCGGATTCAATTGCGGCAGTGACGTCACAGAGGCCCCGCCCCGCCCCCACAAGAGCCCCACCGACGTG(0.83)GGGTTG(0.82)GCGGTGGTGGAAGGACTAGGAGTTGGCGCGTGCGTACTGGCGGCCTCTCCCGCACCGACCGGCCTG(0.80)GGCCCCGCCCCCGGGCGTGAGGCGCCCAATGCGCGTGCGCGGCGGCGTCGGCGCCAGTTATTTCTGTCCCGCCCCCCGGCCTCGGCTCTTTCTGCGAGCGGGCGCGCGGGCGAGCGGTTGTGCTTGTGCTTGTGGCGCGTGGTGCGGGTTTCGGCGGCGGCTGAGGAAGAAGCGCGGGCGGCGCCTTCGGGAGGCGAGCAGGCAGCAGTTGGCCGTGCCGTAGCAGCGTCCCGCGCGCGGCGGGCAGCGGCCCAGGAGGCGCGTGGCGGCGCTCGGCCTCGCGGCGGCGGCGGCGGCAGCGGCCCAGCAGTTGGCGGCGAGCGCGTCTGCGCCTGCGCGGCGGGCCCCGCGCCCCTCCTCCCCCCCTGGGCGCCCCCGGCGGCGTGTGA

PTDSS1 (NM_014754.3)

CCCTCTGCTCCCAGCCTTTGCTGGGCGCCAGACCCGGCTTTGCCGTCCGGCTATTAGCCTACTGTGGCTAGTCACCCCCGGGGTCCCGGCCTTCTCGGGCTGGGGCCGCCGCCACCGCGGCAGGACG(0.83)GGGAGGCGGGCC

TCEA1 (NM_006756.4)

AGCCGGAAGCCACGCCTGCCCACTAGCCCGACGCCCGCCTGGCGGGAAC**ATG(0.85)**GGCTCGCCCCTCACCAGCGATCTGCAGTCAGTTGGTAGCGCCTGCACGTCGCGCGCGGTGTTCGATTGTCGCTGCCTGGGGAGGAGGAGCCGGAGCCGCCGCCGCCGCCGCCGCCGCCGCGGGCTTCGTTCGTAAGGAAG(0.82)GGGGCCTAGGCCCGGGCCTGCGGTGGTGGGGGTTGCTGCGCGCCGGGGGTCGCTCCTGCTGTGTCTTCCGCTCCAGCTTCGCCCACTTCCCCTTGCCAGCGGGGTGGGCGCGGAGAAGACCTGCCGGAGCC

PCNA (NM_002592.2)

GGATGGCCGGAGCTGGCGCCCTG(0.85)GTTCTGGAGGTAACCGGTTACTGAGGGCGAGAAGCGCCAC
CCGGAGGCTCTAGCCTGACAAATGCTTGCTGACCTGGGCCAGAGCTCTTCCCTTACGCAAGTCTCAGC
CGGTCGTCGCGACGTTCGCCCGCTCGCTCTGAGGCTCCTGAAGCCGAAACCAGCTAGACTTTCCTCCT
TCCCGCCTGCCTGTAGCGGCGTTGTTGCCACTCCGCCACC

FDPS (NM_002004.4)

GGGAACTACTCGACCCACAGAGCCGATCGCGGAGCGGATTCTGCTTTTAGGAGTACCCGCCAACAAG(
0.80)CGGGACCGAGCAGGAATCCGTATCTGGGAACAGG

PSMD2 (NM_002808.5)

GCAGCGGGCCGGCAGTG(0.81)GCGGCGGAG

HMGA1 (NM_145899.3)

CTTTTTAAGCTCCCCTGAGCCGGTGCTGCGCTCCTCTAATTGGGACTCCGAGCCGGGGCTATTTCTGGC
GCTGGCGCGGCTCCAAGAAG(0.83)GCATCCGCATTTGCTACCAGCGGCGGCCGCGGCGGAGCCAGG
CCGGTCCTCAGCGCCCAGCACCGCCGCTCCCGGCAACCCGGAGCGCGCACCGCAGGCCGGCGGCCG
AGCTCGCGCATCCCAGCCATCACTCTTCCACCTGCTCCTTAGAGAAGGGAAG

KPNB1 (NM_002265.6)

CCCTCCCTGCGCGCCGCCTCTCACTCACAGCCTCCCTTCCTTCTTTCTCCCTCCGCCTCCCGAGCACCA
GCGCGCTCTGAGCTGCCCCCAGGGTCCCTCCCCCGCCGCCAGCAGCCCATTTGGAGGGAGGAAGTAA
GGGAAGAGGAGAGGAAG(0.80)GGGAGCCGGACCGACTACCCAGACAGAGCCGGTGAATGGGTTTGT
GGTGACCCCCGCCCCCCACCCCACCCTCCCTTCCCACCCGACCCCCAACCCCCATCCCCAGTTCGAGC
CGCCGCCCGAAAGGCCGGGCCGTCGTCTTAGGAGGAGTCGCCGCCGCCGCCACCTCCGCC

DYNLL1 (NM_001037494.2)

CTTCGGGCGCTGACAGG(0.84)GAGAGCCTG(0.85)GGGCCGGGCCGTGTGGATGCCATCCCCGAGCGC
GGTTCGCGCTCGGCTGAGGCGCTGGACAAGTGGCTTGGGCTCCCGCGCCTCAGTTTCTCTCTGTGGCG
CCGCCTACCTCACAGACTTGTGAGCACTCACTGACGTGGGTAGCGCCCAGGGCCTGCGGGGCGCAGG

AGAGCTG(0.80)GAGTCAGGCGGAGACCGCAGGCTGACCCCGCAGCGGCCGGGCTGTCGCGGCCCCC ACCTCAGGTAACC

RUNX (NM_001024630.4)

ATTCGCCTCACAAACAACCACAGAACCACAAGTGCGGTGCAAACTTTCTCCAGGAGGACAGCAAG(0.81)AAGTCTCTGGTTTTTAAATGGTTAATCTCCGCAGGTCACTACCAGCCACCGAGACCAACAGAGTCAT TTAAGGCTGCAAGCAGTATTTACAACAGAGGGTACAAGTTCTATCTGAAAAAAAAAGGAGGGACT

MRPL3 (NM_007208.4)

CAGAGAGCATC(0.82)GGCCGGCGACCGTTCCGGCGGCCATT(0.84)GCGAAAACTTCCCCACGGCTAC TGCGTCCACGTGGCGGTGGCGTGGGGACTCCCTGAAAGCAGAGCGGCAGG(0.83)GCGCCCGGAAGT CGTGAGTCGAGTCTTCCCGGGCTAATCC

SPP1 (NM_001040058.2)

AGCAGCAGGAGGAGGCAGAGCACAGCATC(0.84)GTCGGGACCAGACTCGTCTCAGGCCAGTTGCAG CCTTCTCAGCCAAACGCCGACCAAG(0.83)GAAAACTCACTACC

SNRPD2 (NM_004597.6)

GCGTCCCAGAGGGAAGAGGGGCGGTGCCTGTCTCCGAAGCGAGGAGCGGATTGGCTTCACGGAGGC CGCGCCCCCGAAGTCACG(0.83)GCCGGCGGGCATTTGGAAATAGCTTGTCATTTCCCTCCTTCAACCT GTACCATTCTTCAAGAAACGGCGCCAACGGAAGTGGGTCGCAGGAAGAGGAAGTCCCGCCTCTCTCT CCTCAGGCAGCAGCAACGCGGAGGAAACGGGAGTGAACGGAGAGCGTAGTGACCATC

UCHL3 (NM_001270952.2)

GCTCGGCAAGGCTCGGCTCGGAAGAGTCCAAGCGTGAGGGGAGAGGGCTGTGGATTCAGATACTGTT TTTCTCCCGAAATAGGAAATTGGCTTATTTTTTTCTCCTCGGCAGCATCTTAATTTAAAATATGACACTTG ACCTACGGCCCTGCACGGAGCGGTTAAGAGG(0.80)GTCACCAACCAGTTTCTTAAACAATTAGGTCTA CATCCTAACTGGCAATTCGTTGATGTATATGGA
